# Gradient Hydrogels—Overview of Techniques Demonstrating the Existence of a Gradient

**DOI:** 10.3390/polym14050866

**Published:** 2022-02-23

**Authors:** Natalia Zinkovska, Miloslav Pekar, Jiri Smilek

**Affiliations:** Faculty of Chemistry, Brno University of Technology, Purkynova 464/118, CZ-61200 Brno, Czech Republic; xczinkovska@fch.vut.cz

**Keywords:** gradient hydrogels, concentration gradient, physico-chemical techniques for gradient proof, intelligent supramocelular gels, biopolymer 3D gels

## Abstract

Gradient hydrogels are promising future materials which could be usable in tissue engineering (scaffolds), pharmaceutical (drug delivery systems with controlled release) and many others related disciplines. These hydrogels exhibit a more complex inner (gradient) structure (e.g., concentration gradient) than simple isotropic hydrogel. Gradient-structured hydrogels could be beneficial in, for example, understanding intercellular interactions. The fabrication of gradient hydrogels has been relatively deeply explored, but a comprehensive description of the physico-chemical techniques demonstrating the existence of a gradient structure is still missing. Here, we summarize the state-of-the-art available experimental techniques applicable in proving and/or describing in physico-chemical terms the inner gradient structure of hydrogels. The aim of this paper is to give the reader an overview of the existing database of suitable techniques for characterizing gradient hydrogels.

## 1. Introduction

Gradient hydrogels are a type of special hydrogel with a more sophisticated and controlled architecture [1,2]. As the name indicates, they do not have an isotropic structure but inherently contain a property (or properties) that change in space. These may include structural, compositional, or functional properties. Gradients can be made either as steps or non-abrupt, i.e., exhibiting a continuously changing slope. Naturally, it is of crucial importance to verify the formation of the desired gradient.

The introduction of a gradient represents one of the techniques for tailoring hydrogel properties to various applications [3,4,5]. Often the gradient structure is inspired by materials found in nature and intended for medical applications [6,7,8]. Recently, we conducted a review of recent advances in the preparation of gradient hydrogels where such aims were also reported [9]. In a response to that paper, an anonymous reviewer suggested that we also perform an overview of instrumental techniques used to prove the existence of gradients and to characterize them. This was the motivation for the current paper, which thus complements the previous review. We focused on relatively recent works, preferably not older than about five years, in order to provide an overview of the current state of the art in this area.

The reported techniques were grouped under several headings, which are reviewed separately. Usually, authors use multiple techniques to characterize their hydrogels from different points of view, and sometimes gradient observation or verification was performed with more than one method. In those cases, we report on the basic principle of gradient formation at the first appearance of the work referred to. A detailed overview of related techniques is provided in the Supplementary Materials (Table S1).

## 2. State of the Art

### 2.1. Visual Inspection

Visual inspection is the simplest, most straightforward method with low resolution. It usually requires coloring with a suitable dye, which itself should not create a “false gradient”. Its application is self-evident, and we will thus report only a few examples randomly selected from recent literature.

Gharazi et al. prepared hydrogels consisting of several distinct zones progressively differing in their stiffness [10]. In other words, a steep mechanical gradient was formed. The zones were covalently crosslinked. For the preparation of the soft and stiff zones, two different materials with controlled crosslinking density were used. To visualize zones with different composition and stiffness, the precursors of different zones were colored with different dyes (rhodamine 6G, malachite green), and the natural appearance of some zones (transparent versus white) was used. The result was a cylindrical length of hydrogel with, for example, four clearly distinct colored parts.

A similar approach was used by Cho to visualize their multi-compartmental hydrogel microparticles [11]. The compartments differed in their compositions and were colored by Alexa fluorescent dyes added to the gel precursor solutions.

**Figure 1:**
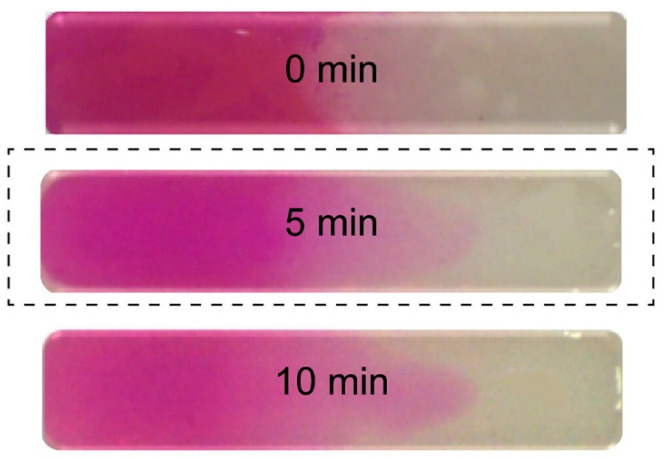
Formed uniform gradient (the optimal time for the uniform mixing of solutions was observed to be 5 min) [12].

A continuous, progressive gradient was formed by Cross et al. by adding two pre-polymers in the opposite sides of a flat rectangular well [12]. A gradient was formed by mutual diffusion at the contact line in the well and fixed by UV-initiated crosslinking. One of the prepolymers was colored (rhodamine B), and the diffusion of the dye was supposed to follow the gradient-forming diffusion. The gradient was thus indirectly but successfully visualized, as illustrated in Figure 1.

**Figure 2:**
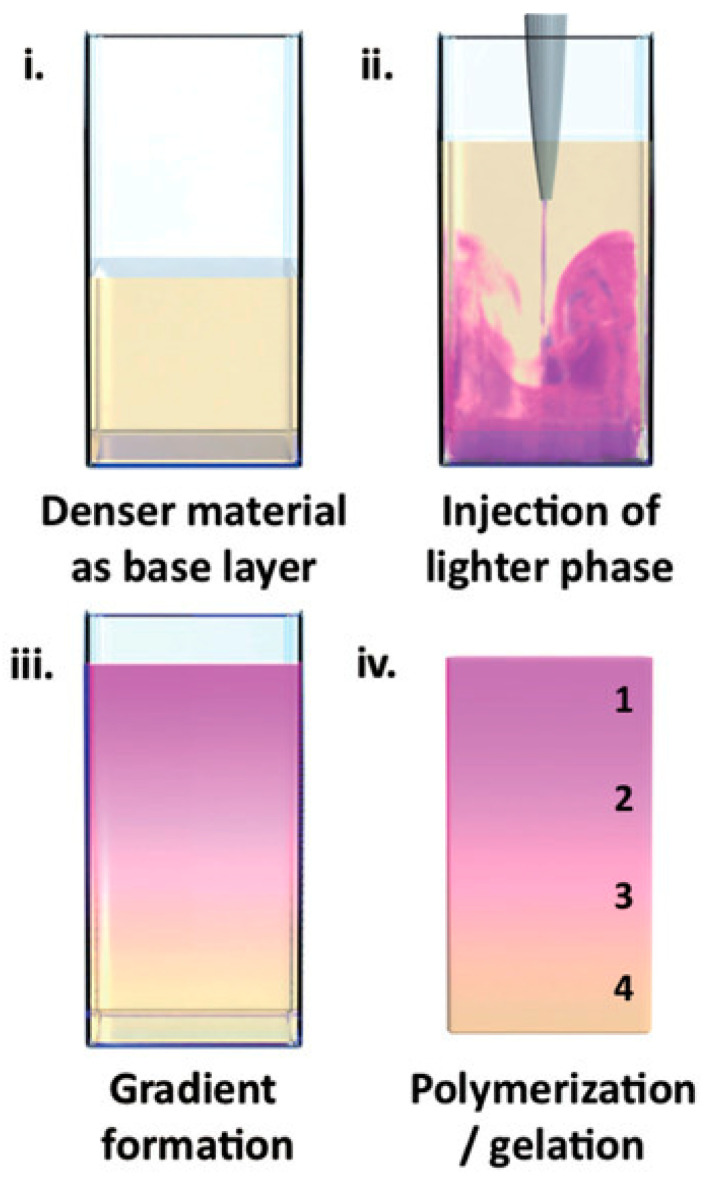
An example of gradient formation (the gradient is preserved by gelling or polymerizing the material) [13].

Li et al. employed a universal physical principle to prepare gradient hydrogels that could be applied broadly across different material systems [13]. Specifically, they used buoyancy, the upward force generated on liquid injected into a denser fluid phase via a single injection. The gradient formed by buoyancy was then fixed by gelation. This experimental approach was applied for the preparation of gradient hydrogels based on polysaccharides (agarose, gelatin methacryloyl, gellan gum) as well as acrylate polymers. To visualize the resulting gradients, one of the liquids was dye-labeled, or colored. For example, agarose was tagged with rhodamine B and injected into a solution of native agarose, or rhodamine B was added to *N*,*N*-dimethylacrylamide, in which it is soluble, in contrast to lauryl methacrylate, the second component forming the acrylic gradient hydrogel. Either a steep or continuous color distribution across the gradient borders was observed, as illustrated in Figure 2.

### 2.2. Chemical Analyses

Chemical analyses consist of spectroscopic techniques that are employed at various levels of distance, spatial resolution, or scanning. They can be applied if the gradient is formed due to gradient changes in chemical composition to which spectroscopy is sensitive. The benefits and limits of chemical analysis methods are summarized in the Supplementary Materials (see Table S2).

A crude application using X-ray photoelectron spectroscopy (XPS) and fluorescence spectroscopy (FLUO) was described by Fan et al., who prepared two-layered hydrogel sheets with a thickness of about 0.5 mm [14]. The two layers were of different crosslinking densities, and were created by adding graphene oxide into their precursor, which served as a UV absorber in the UV-curing process. UV-irradiation was applied to one side of the sheet only, and the irradiated side thus possessed higher density. Raman spectra were taken on both sides of the sheet, and differences in the degree of carbon order were detected, which served as indirect proof of a dual (two-sided) gradient. Similarly, the two hydrogel sides were examined by XPS spectroscopy. The results were rather confusing, and were discussed only briefly. No differences in nitrogen, oxygen, or carbon contents were found with respect to the two sides, which was interpreted as indicating the presence of a similar degree of crosslinking. Due to the much higher polymeric chain density on the UV-irradiated side, detected by fluorescence microscopy, than on the LD side (low-degree side), the authors concluded that “this side possesses a higher crosslinking density (i.e., a higher number of crosslinking bonds per unit volume of the gel) than the other side”. The difference between the crosslinking degree and density is unclear. 

A more sophisticated technique, based on Raman spectroscopy (RAMAN), was reported by Kim at al., who used it to explicitly characterize the spatial profile of their hydrogels [15]. The gel was made from methacrylated gelatin using UV-crosslinking through a gradient photomask. In this way, a linear stiffness gradient was developed. In fact, Raman spectroscopy was used to follow the spatial profiles of amide groups in the hydrogel. A hydrogel sample with dimensions of 11 mm × 11 mm × 0.5 mm was placed on a coverslip and mounted on the stage of a confocal Raman microscope to verify a vertical gradient. The microscope was focused at 50 µm above the coverslip surface, i.e., inside the hydrogel. Two amide groups were identified, and both vertical and horizontal scans were realized at several lines. No changes in the amount of amide were detected parallel or perpendicular to the gradient. Consequently, chemical analysis was not appropriate for determining or confirming a gradient in the studied material, and it was shown that stiffness gradient was not accompanied by a concentration gradient (of the polymeric precursor), but only by increased crosslinking density.

**Figure 3:**
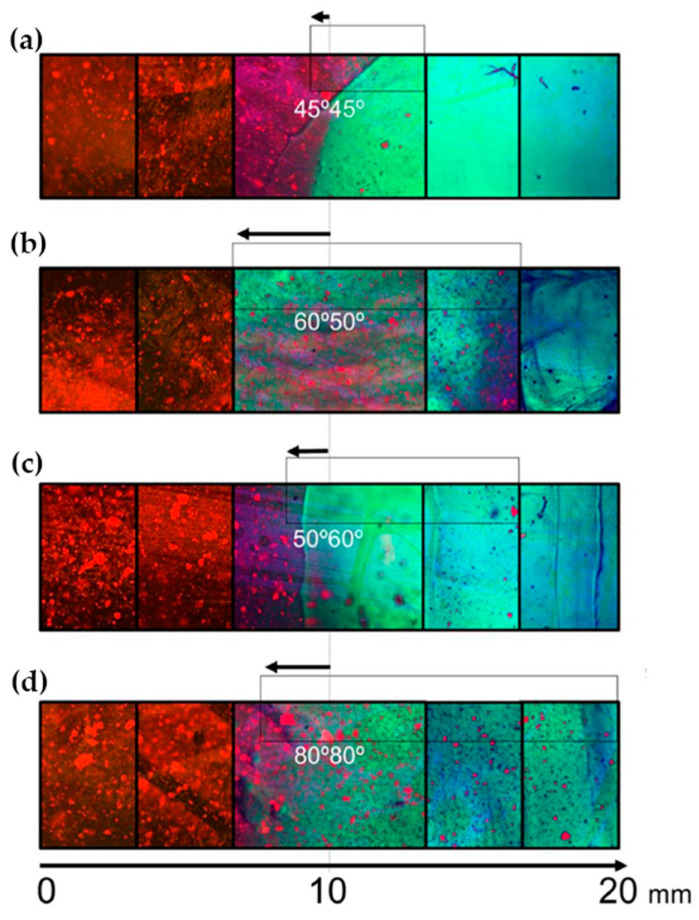
Gradients patterned by HAp-controlled dispersion resulting from different gel viscosities/temperatures tracked by fluorescence microscopy using a GG modified with FITC (green) and the fluorescence of alizarin red-stained HAp (red) (**a**) 45–45 °C, (**b**) 60–50 °C, (**c**) 50–60 °C, and (**d**) 80–80 °C [17].

**Figure 4:**
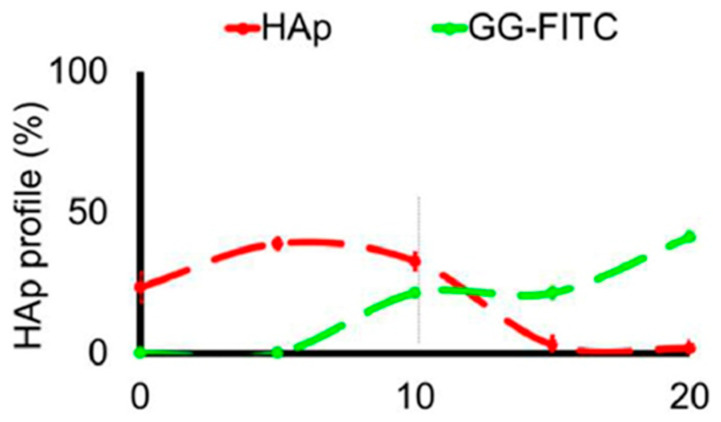
The profiles of HAp and GG-FITC distributions through the 3D structure for mixing temperatures 45–45 °C [17].

Fluorescence spectroscopy is often used in the form of labeling a gel-forming component with suitable fluorescent moieties. Using gelatin labeled with fluorescein isothiocyanate, Gorgieva et al. fabricated a hydrogel with a porous gradient structure ranging from 100 to 1000 µm [16]. The label enabled visualization of the porous structure under a fluorescence microscope. Microscopic images showed different parts of the hydrogel illustrating pores of different sizes. The gradient was thus demonstrated with selected points, not by a continuous strip. Canadas et al. generated 3D large-ranged gradient structures by controlling the spatiotemporal dynamics of mixing two gellan gum gels at two different temperatures [17]. One of the gels was filled with hydroxyapatite microparticles (Hap). To observe the formed structures by means of fluorescence microscopy, the pure gellan gum was covalently modified with fluorescein isothiocyanate, while the microparticles were stained with alizarin red. After achieving the target temperature of the solutions, the first layer of the pre-gel solution was injected into a mold, and the second layer was injected on the top of the first gel. The mixing process of the layers was tracked by fluorescence microscopy. Microscopic images showed the gradient profiles of the hydroxyapatite microparticles and the fluorescein-labeled gellan (Figure 3). The observed profiles demonstrated the presence of a continuous gradient for both components in the gel structure (Figure 4). While the mixing of the layers mainly occurred at the interface and the interpenetration into the adjacent gel was generally low, the gradient patterning largely depended on the temperature variation between the first and the second layer.

Liu et al. [8] cut slices of fabricated hydrogel (250 μm in thickness) along the gradient axis and loaded them onto a glass slide for fluorescence imaging. To this end, the polyethylene glycol precursor was labeled by rhodamine, and the fluorescence signal was quantified. The continuous gradient structure was confirmed by a continuous increase in fluorescence intensity from one end of the hydrogel to the other.

Confocal laser scanning microscopy (CLSM) based on fluorescent dyes was included in a set of methods applied by Fan et al. [14] to characterize their two-layered hydrogels. A solution of two colorants was used, and the whole hydrogel was immersed in it. Sodium fluorescein bonded to oppositely charged chains of poly(*N*,*N*-dimethylaminoethyl meth-acrylate), the hydrogel-forming polymer, whereas rhodamine 6G bonded to graphene oxide due to π-π stacking. The former dye gives green fluorescence, while the latter gives red fluorescence. The cross-section of the hydrogel was then observed under a fluorescence microscope. Green fluorescence was emitted from a bounded area on the UV-irradiated side of the sample whereas red fluorescence was distributed much more uniformly along the cross-section. This confirmed a step gradient with the density of the polymeric chains.

Ko et al. formed a concentration gradient of bovine serum albumin in methacrylated gelatin hydrogel [18]. First, a base hydrogel without albumin was created by UV-crosslinking of an appropriate prehydrogel solution. Then, a set of prehydrogel solutions containing increasing concentrations of albumin was overlaid and crosslinked sequentially. Albumin was first conjugated to a green-fluorescent dye to enable visualization of the albumin concentration gradient by fluorescence microscopy. A continuous change in green coloring was then observed along the 2.5 mm sample length, which could be transformed into a graphical fluorescence intensity–distance profile.

Shao et al. used micro-FTIR (micro-Fourier transform infrared spectroscopy) to detect the chemical structure of the cross-section of polyampholyte-based hydrogels with a gradient compositional and porous structure [19]. The hydrogel was synthesized by the copolymerization of sodium p-styrenesulfonate and methyl chloride quarternized *N*,*N*-dimethylamino ethylacrylate using 2-ureidoethyl methacrylate as a physical cross-linker. By using a reaction mold composed of a glass plate on the top, a silicone spacer in the middle, and the Teflon plate at the bottom, and due to the different hydrophobicities of the sides, the composition in the copolymer gels showed a gradient distribution. With increasing distance from the Teflon side, the color of the peaks steadily changed from blue to red, which corresponded to a gradual change in the signal from weak to strong. This confirmed a gradient distribution of composition in the hydrogels along the observed distance of 300 μm.

### 2.3. Structural Analyses

As expected, electron microscopy techniques, especially Scanning Electron Microscopy (SEM), are among the most widely used means for checking gradients when these consist of structural (porous) features. Hydrated samples like hydrogels cannot be directly observed, and are usually lyophilized before observation or frozen and observed in solidified form (cryo-SEM, for example). The benefits and limits of structural analysis methods are summarized in the Supplementary Materials (see Table S2). The structure of lyophilized samples can differ significantly from the sample structure in the hydrated state—in fact, only a dried porous structure is observable, which is believed to represent the hydrated structure. Cryo-techniques are more common in the hydrogel area, but even here, artifacts can be formed if the freezing process is not performed properly [20].

**Figure 5:**
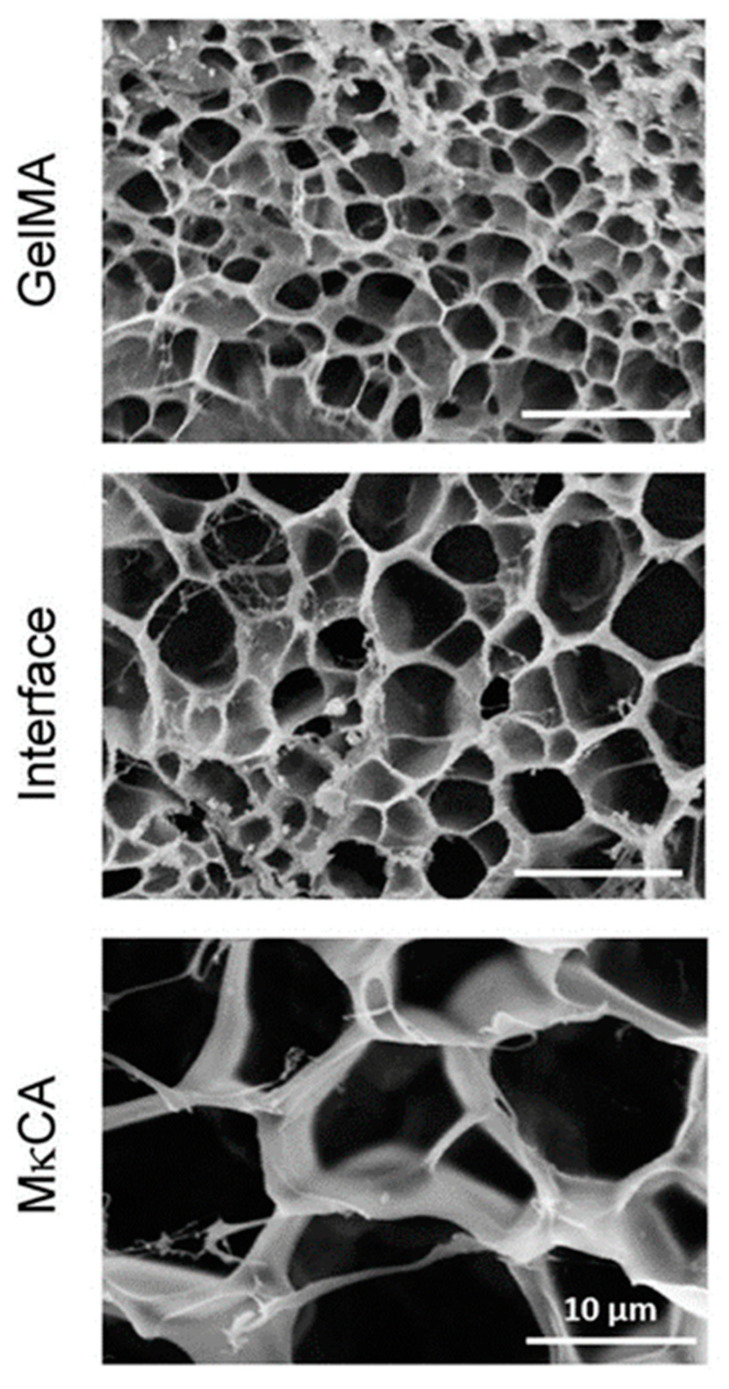
Scanning electron micrographs of gradient hydrogels (GelMA-MκCA). A significant change in pore size distribution was observed [12].

Cross et al. [12] used the cryoscopic fracture of samples frozen in liquid nitrogen to open the cross-sections of their hydrogels. Samples were observed after lyophilization, i.e., in the dry state. The authors observed a distinct change in pore areas when moving from the region formed from methacrylated gelatin to the region based on methacrylated kappa-carrageenan, the two pre-polymers used to create the gradient by their inter-diffusion before irradiation crosslinking (see Figure 5). The average pore size area changed from about 4 through 17 at the interface, and up to 75 µm^2^. Thus, only three points within the gradient hydrogel were observed, and not a gradual change in porosity.

Using SEM, Fan et al. [14] visualized the cross section of their two-layered hydrogel. Images were taken at two points and showed the porous structure of the sample to have a higher density on the irradiated side in comparison with the non-irradiated side. The samples were prepared by freeze drying, fracturing in liquid nitrogen, and gold sputtering on the fracture surface.

Gorgieva et al. [16] focused primarily on the gradient in the porous structure. They visualized both the surfaces and the cross-sections of their lyophilized (i.e., dry) and gold-sputtered materials. The cross-section was exposed by simply cutting the sample with a razor blade. Pores across the whole cross-section of approximate thickness 3 mm were sufficiently large to be observed at rather low magnification. The pore diameter ranged from about 1 mm near the surface skin up to about 100 µm close to the bottom side of the hydrogel. Thus, a gradient of sufficiently large pores could be observed throughout the whole hydrogel sample at the same time.

Hydrogels with stiffness gradients were prepared by Kim et al. [15] from methacrylated gelatin using photomask-assisted UV-crosslinking. SEM showed that the stiffness was inversely correlated with pore structure. Samples for SEM were prepared by flash-freezing and freeze-fracturing using a commercial system, followed by sputtering with platinum. Several images were captured along the hydrogel lengths; three images are shown in the paper, with the pores’ dimensions being on the order of microns. 

Another example of using scanning electron microscopy as a characterization technique for gradient structures was documented by Scaffaro et al. [21]. Three-layered PLA/PEG scaffolds with a pore size gradient were created by melt mixing using sodium chloride NaCl and polyethylene glycol as porogen agents. The choice of an appropriate NaCl grain size filled in the polymer matrix enabled control of the pore architecture of every single layer and subsequent prediction of the pore size distribution in the complex three-layered structure, while the use of PEG enhanced pore interconnections. The morphologies of both monolayered and multi-layered structures were evaluated using SEM before and after growth of the cell culture. Conventionally, samples were frozen by liquid nitrogen and then sputter coated with gold. Observation of the multilayered samples confirmed the pore size distribution, where the mean pore size of every layer in the scaffold corresponded to the pore dimensions of the respective monolayer. Three continuously connected layers (0.5–0.7 mm in thickness) of different porosity were observed over a thickness of 2 mm.

**Figure 6:**
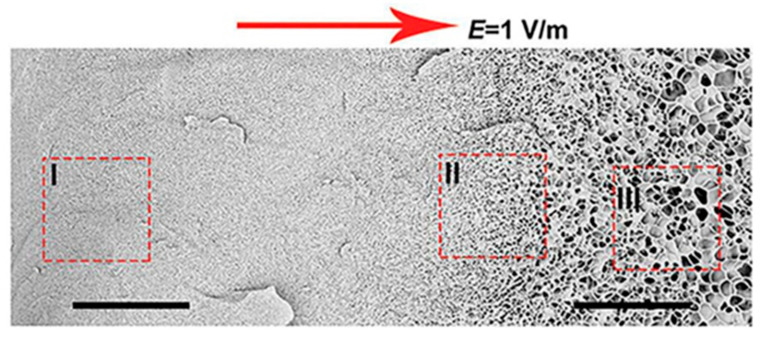
Scanning electron micrographs of freeze-dried sample with a large-ranged gradient structure along the direction of the electric field [22].

Yun Tan presented new temperature-responsive hydrogels with bionic large-ranged gradient structures [22]. Hydrogels were fabricated by the copolymerization of the hydrophilic monomer hydroxyethyl acrylate (HEA) and *N*-isopropylacrylamide (NIPAM) in a dispersion of laponite platelets using the electrophoretic method. The morphology of the prepared freeze-dried bionic gradient hydrogels was analyzed after gold sputtering. SEM images (see Figure 6) revealed a long-ranged gradient porous architecture along the direction of the electric field due to the crosslinking effect and the migration of laponite particles towards the anode during the copolymerization of monomers. The pore network dimensions gradually increased from the anode side to the cathode side over the observed sample length of about 0.6 mm.

Ko et al. [18] developed a layer-by-layer technique followed by photo-crosslinking to generate gradient gelatin methacryloyl hydrogels. The individual layers were constructed from prehydrogel solutions of varying concentrations. This resulted in differences in the porous structure of individual layers with perhaps a smooth change across the inter-layer region, as demonstrated by SEM images of freeze-dried and iridium sputter-coated samples. The images of individual layers showed smaller showed some cracked layers that were not easily interpretable but showing a decrease in pore size in the direction of increased pores with increased prehydrogel concentration. The image of the whole sample (length about 1.3 mm) concentration. In addition, thicker pore walls were observed with increased concentration. 

Atomic force microscopy (AFM) is a relatively modern microscopic technique enabling the local assessment of mechanical properties. Kim at al. used AFM to check the stiffness gradient along their hydrogels (thickness of 15 mm) [15]. Indentation by 200 µm triangular cantilever tips was applied at 1 mm intervals. Young’s modulus, determined from the AFM data, showed a linear increase along the hydrogel thickness from about 4 to about 12 kPa. The AFM technique thus enabled the real gradient to be followed in sufficient spatial resolution, which in essence demonstrated its continuity. In fact, AFM combines microscopic and mechanical analyses, the latter being the subject of the next section.

Atomic force microscopy was also used by Xu et al. [23] to detect the aggregation morphology of BSNF (beta-sheet rich silk nanofiber), used as a mechanical reinforcement blended with amorphous silk nanofiber solution to form hydrogel matrices. Beta-sheet building blocks migrated to the anode along the applied electric field and resulted in a gradient distribution in the formed hydrogels. AFM was applied on four parts of the hydrogel, equally divided along the electrical field direction. Higher contents of beta-sheet blocks near the anode led to the formation of more aligned layers, which resulted in the continuous gradient of such aligned structures along the sample and respective changes in mechanical properties. The gradual increase in beta-sheet content from the area near the cathode to that near the anode was further confirmed by XRD (X-ray powder diffraction) patterns, another structure-revealing technique, and FTIR spectra taken at several selected points along the electric field, i.e., in combination with the chemical analysis technique.

An interesting approach (a novel welding method) for preparing highly anisotropic (cellulose) hydrogels with a programmable-oriented polymer structure and unique compression/tensile properties was developed by Mredha et al. [24]. The oriented (gradient) structure of these anisotropic hydrogels with hierarchical orientation was studied using polarizing optical microscopy (POM), scanning electron microscopy (SEM), and atomic force microscopy (AFM). Images from polarizing optical microscopy confirmed that the anisotropic structure was retained after the welding process. Both parallel-laminate as well as orthogonal-laminate hydrogels exhibited a strong interference color at a 45° angle, which is characteristic of birefringence. In contrast, concentrically rolled hydrogels showed a clear cross-shaped dark pattern, indicating their concentric orientation, whereas axially rolled hydrogels exhibited irregular dark patterns owing to their axially oriented polymer structures. SEM analyses indicated that the hydrogel films were completely integrated by an isotropic interfacial region. These results were further confirmed by AFM.

**Figure 7:**
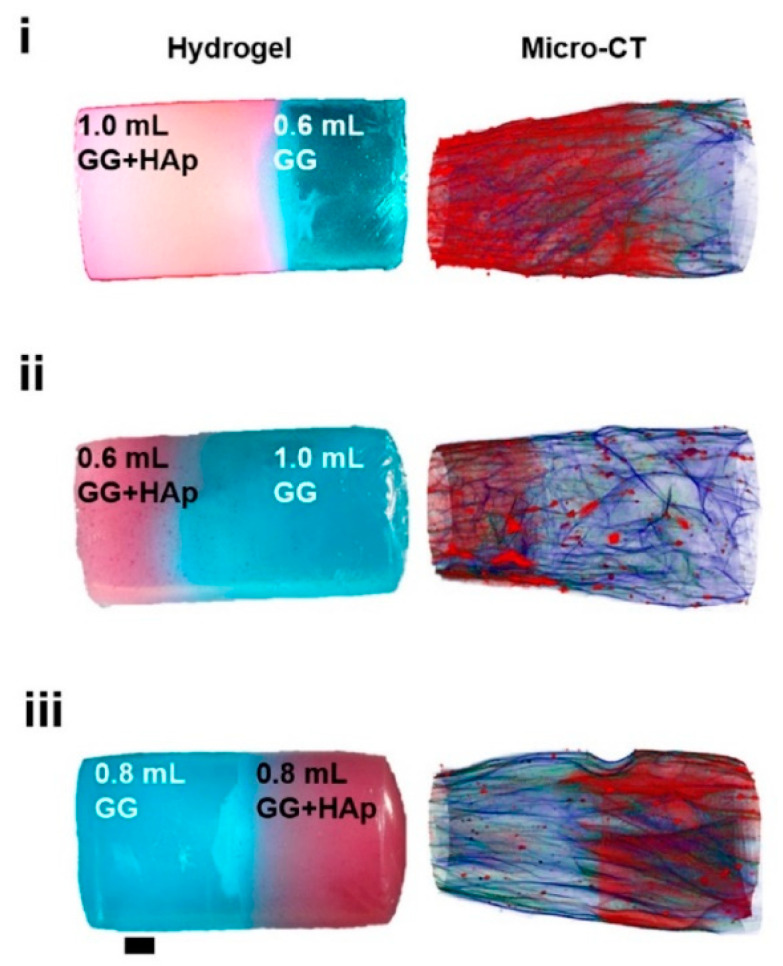
3D images of hydrogels with different solution volume ratios and different HAp distribution and polymeric architecture reconstructed by micro-CT [17].

A rather rare technique in hydrogel research—computer microtomography by X-rays (see Figure 7)—was used by Canadas at el. after the freeze-drying of their gellan-based hydrogels filled with microparticles [17]. This technique enabled the polymeric and microparticle profiles along the structure axis to be observed as well as the pore interconnectivity and porosity distribution to be determined. The pore size decreased with increasing amount of microparticles, while the interconnectivity, and wall thickness were barely affected. In other words, no significant gradient in porosity was observed.

### 2.4. Mechanical Analyses

Traditional methods (e.g., oscillatory measurements, dynamic mechanical analysis) of mechanical analysis may be too crude to detect fine gradients. Specific approaches can be found to address this problem. On the microscopic level, mechanical testing can be realized using AFM, as reported in the previous section for structural evaluations. 

Cross et al. [12] adjusted a standard mechanical testing machine by applying an insert with a conical (1 mm) head. This arrangement enabled six readings to be taken along the hydrogel gradient and the compression modulus to be determined (COMPRES). The modulus showed a nonlinear decrease along the 10-mm-thick gradient hydrogel from about 7 to about 2 kPa for the native hydrogel and from about 8 to about 4 kPa for the hydrogel added with silicate nanoparticles. Fitting the six-point data gave a rough indication of a continuous change in the stiffness gradient. The error bars of some data points were rather wide, suggesting that exact gradient reproducibility is not an easy task.

Scaffaro et al. [21] investigated their three-layer hydrogel scaffolds by compression testing and compared the results with the compression of corresponding monolayered materials. The tests were performed both in dry and wet states. The stress–strain curve of the dry three-layered material showed in the very first part three small but visible plateaus in contrast to the mono-layered sample. The authors hypothesized that this was a demonstration of the collapse of one layer at a time, which started with the weakest layer. No three plateaus were not observed in the wet state; however. The compression elastic modulus of the three-layer sample was comparable with the modulus of its weaker layer, both in dry and wet states.

In a similar way, Shi et al. investigated the mechanical properties of their three-layer hydrogels prepared from dopamine-modified alginate and chitosan–hydroxyapatite [25]. The layers differed in the ratio of these two components. Samples of both separate layers and the gradient sample were subjected to unconfined compression testing using a universal testing machine. The stress–strain behavior and compression modulus at 10% strain of the scaffolds were presented. The compression modulus increased with higher proportions chitosan–hydroxyapatite, i.e., from the top to the bottom layer of the gradient material. The stress–strain curve and the compression modulus of the gradient hydrogel were between those measured for the middle and bottom layer; the curve of the layered product showed no special features, pointing to its gradient character. Mechanical testing thus did not confirm the existence of a chemical gradient directly, but showed differences in the layers’ components and the overall outcome of their combination.

Su and colleagues [26] investigated the mechanical properties of bilayered anisotropic gradient poly(vinyl alcohol)/hydroxyapatite composite hydrogels that were obtained by the combination of a directional freeze–thaw process and the electrophoresis method. The freeze–thaw step first offered the possibility to prepare materials with a long aligned porous structure, while further freeze–thaw cycles led to the reforming of an arranged structure due to the breaking of pore walls; thus, the pore dimensions increased. During the electrophoresis part, hydroxyapatite particles were synthesized within the formerly prepared matrix of poly(vinyl alcohol)hydrogel, resulting in a bilayered structure, i.e., a region containing particles and a region without particles. Samples were cut parallel to the freezing direction into dumb-bell-shaped specimens for tensile tests and cylindrical shaped specimens for compression tests. The samples were taken from three different parts, namely from each of the two layers differing in the presence of the hydroxyapatite particles and from their interface.

Stress–strain curves confirmed that hydrogels subjected to the same number of directional freeze–thaw cycles had distinct tensile properties in different regions. In general, the initial tensile modulus gradually increased from the region without particles to the region containing particles, while elongation at the break decreased in the same direction. The initial tensile modulus, as well as the breaking strength, gradually increased with increasing number of directional freeze–thaw cycles. Furthermore, during the tensile tests (TENSILE), the interface was not broken at the border line, which demonstrates a relatively high interlayer binding strength. On the other hand, elongation at break and/or the breaking strength was lowest for the interface region. The initial compressive moduli for gels with the same number of directional freeze–thaw cycles gradually increased from the region without particles through the interface up to the region with particles. As in the previous case, this was not direct confirmation of the existence of a gradient.

**Figure 8:**
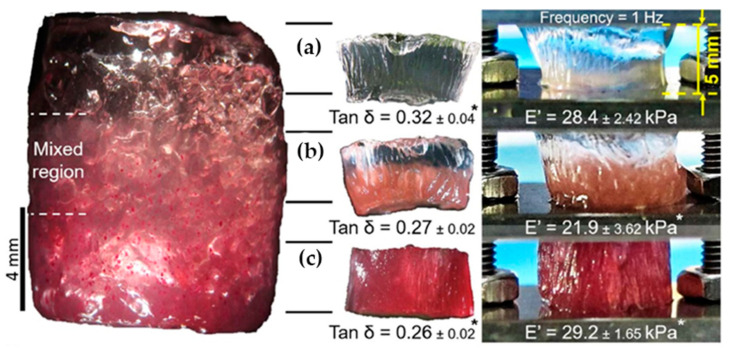
The dynamic mechanical behavior of (**a**) top, (**b**) middle and (**c**) bottom regions of gradient hydrogels together with Young’s moduli and phase angles [17].

In a similar way, Canadas et al. [17] followed the dynamic mechanical behavior (DMA) of the top, middle, and bottom regions of their gellan-based hydrogels (see Figure 8). While the storage moduli were nearly constant in all three regions, the loss angle decreased with increasing filler microparticle content.

**Figure 9:**
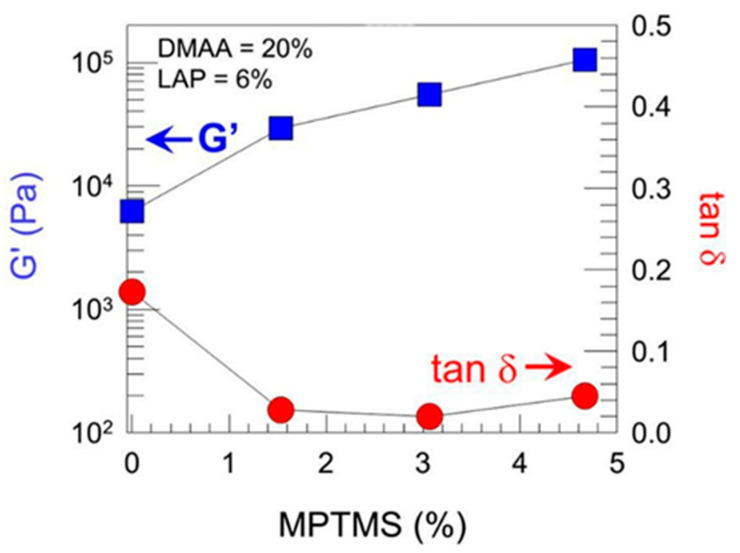
Oscillatory shear rheology of different zones of a soft−stiff hybrid gel, where G′ and the loss tangent tan δ are plotted as a function of MPTMS content with DMAA held constant at 20% and LAP at 6% [10].

**Figure 10:**
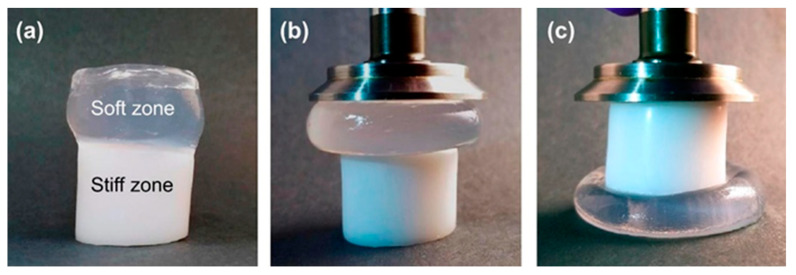
Figures showing the behavior of soft−stiff hybrid gels under compression: (**a**) initial hydrogel before compression, (**b**,**c**) compressed hydrogel by rheological plate geometry [10].

Sharp gradients like those observed for zonal hydrogels designed by Gharazi et al. are relatively easily checkable [10]. The materials of individual (inter-gradient) zones can be separately subjected to mechanical tests. More interesting are tests of the whole material, but care should be taken when interpreting their results, because the overall response is composed of the responses of the individual zones, which can differ considerably. The visual inspection of sample deformation can also be helpful. Accordingly, Gharazi et al. performed compression tests in a standard rheometer in squeeze mode. Hydrogel samples with a thickness of 4–10 mm were compressed at a rate of 10% strain per minute based on the initial thickness, the authors only presented compression curves for the stiff and soft zones separately (see Figure 9). In addition, the response of the gradient two-zone material was visualized by means of manual compression; see Figure 10.

The combined material retained the compression characteristics of the individual zones. Similar behavior was observed in manual stretching.

Indirect proof of a mechanical gradient was presented by Ko et al. [18]. They generated mechanical gradients by stacking layers of different gelatin methacryloyl concentrations, but measured Young’s modulus in the compression of each layer prepared separately as an individual hydrogel. As expected, the hydrogels with increasing concentrations of prehydrogel exhibited higher moduli, ranging from 5 to 80 kPa.

Xu et al. [27] also measured the Young’s moduli of different parts of a nonswellable gradient hydrogel, which was synthetized via the acid-heat treatment of polyamide-based hydrogels to convert hydrophilic amide groups into hydrophobic imide groups. Due to the different diffusion rates of hydrochloric acid from the outside layer to the inside layer of the hydrogel, a gradient distribution of imide groups was achieved. AFM measurement confirmed a gradual decrease in Young’s modulus from the surface to the inside of the IPAM NC hydrogel.

Li et al. measured the contact modulus along the longitudinal axis of their gradient hydrogels using spherical indentation mapping (INDENTAT) [13]. The contact modulus of a stainless-steel sphere 3 mm in diameter was determined while indented along the gradient in 0.5 mm steps. Almost twenty measuring points were thus located along the gradient and showed the formation of either steep (an S-shaped modulus-position curve) or approximately linear gradients, depending on the parameters of the hydrogel preparation methods. Within the space resolution of the indenter, the gradient could be directly and, in principle, continuously monitored and verified.

Anisotropic multilayered hydrogels prepared by Mredha et al. [24] were studied by means of both tensile and compression tests. The tests were not designed to check the existence of gradients, but to demonstrate specific mechanical properties of their materials. Parallel-laminate hydrogels showed very strong mechanical anisotropy. Along the parallel-laminate direction, the maximum Young’s moduli were 4–12 times higher than those along the perpendicular direction. In contrast, orthogonal-laminate hydrogels showed orthotropic tensile properties which were similar in the parallel and perpendicular directions.

### 2.5. Miscellaneous

Gradient hydrogels are often fabricated with regard to their potential use in applications in tissue engineering. In such applications, the hydrogel’s effects on cells are important. Cross et al. [12] encapsulated human mesenchymal stem cells within their gradient hydrogels, which were therefore prepared from solutions in culture media instead of deionized water. The cells were inspected after one and three days at three different locations corresponding to different parts of the gradient. Distinct changes of cell morphology were observed. In the gelatin-rich part, characteristics of osteoblasts in bone were detected, whereas in the carrageenan-rich part the morphology of chondrocytes in cartilage was found. At the interface, both morphologies were present, which was considered proof of the smoothness of the gradient. The average cell adhesion area decreased from the gelatin part through the interface to the carrageenan part.

Tan et al. [28] fabricated a laponite-crosslinked gradient hydrogel based on copolymer of (*N*,*N*-diethylacrylamide and (2-dimethylamino) ethylmethacrylate. The laponite concentration was determined using the thermogravimetric analysis (TGA) of samples taken from three different locations of the final material. TGA showed different residual masses of laponite for samples with different of Lap particles at 900 °C. This laponite migration was further confirmed together with the migration of the gel-forming polymer by FTIR spectra taken from the same locations. These three-point characterizations suggested the gradient distribution of laponite and polymer in the hydrogel matrix.

**Figure 11:**
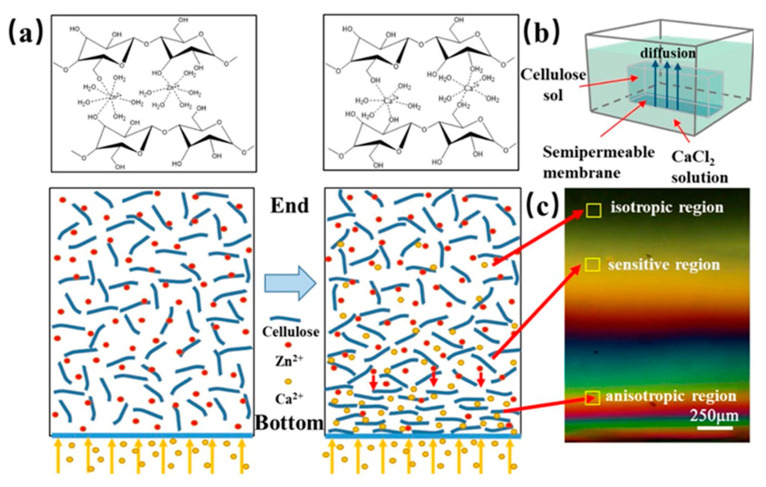
(**a**) Schematic showing the mechanism of preparing gradient hydrogel via the dialysis of cellulose sol in Ca^2+^ solution. (**b**) Schematic illustration of Ca^2+^ diffusion forming gradient hydrogel. (**c**) Polarized images of different diffusion regions of Ca^2+^ in the gel [29].

Kechun Guo et al. used another interesting approach to evaluate the gradient structure of anisotropic cellulose hydrogels [29]. Calcium ions were arranged to diffuse into a cellulose sol causing the gradual orientation of the cellulose chains and forming the cross-linking structure of a gel. At the bottom of the respective mold, the Ca^2+^ concentration and the density of the cellulose chains were the highest, while the concentration of Ca^2+^ at the opposite end was the lowest and the cellulose chains were thus loose and oriented in a disorderly manner. The gradient structure was inspected with a polarizing microscope, see Figure 11. At the bottom, hydrogels exhibited obvious birefringence and the interference was denser. In contrast, at the opposite end, the interference color was hardly observed. The birefringence gradually decreased with the direction of Ca^2+^ diffusion (from the bottom up).

## 3. Conclusions

An ideal technique for proving gradients in hydrogels should allow continuous monitoring of the formed gradient across its full length. In some cases, this is possible; the simplest way would be via the visual inspection of dyed gradients; at a more advanced level, such monitoring could be achieved by taking spatial spectroscopic images. Evidently, the success of this “holistic” approach will be a result of the combination of the dimensional subtleness of the gradient and spatial window and the resolution of the imaging technique.

More often, the hydrogel is sampled across the expected gradient and the “point samples” are analyzed using a suitable technique. Such discrete points are used to reconstruct the gradient and its shape. This approach is most appropriate in the case of steep gradients, when a smaller number of properly located sampling points is sufficient. An indirect alternative can be used in the case of (steep) gradients formed by layering, when the individual layers can be analyzed separately, even without being layered into the final material.

Though hydrogels are three-dimensional materials, perhaps all gradients are two-dimensional. That is, gradients are formed along the (longer) axis and not across the cross-sections. However, the characterization of a potential cross-sectional gradient should be realized by the same techniques applied on cross-cut samples.

## Data Availability

Not applicable.

## Supplementary Materials

The following supporting information can be downloaded at: https://www.mdpi.com/article/10.3390/polym14050866/s1, Table S1: Summary of techniques; Table S2: Overview of characterization techniques; Table S3: List of abbreviations.

## References

[1] Jo H., Yoon M., Gajendiran M., Kim K. (2020). Recent Strategies in Fabrication of Gradient Hydrogels for Tissue Engineering Applications. Macromol. Biosci..

[2] Gadjanski I. (2017). Recent advances on gradient hydrogels in biomimetic cartilage tissue engineering. F1000Research.

[3] Xie W.K., Duan J.J., Li J., Qi B., Liu R., Yu B.Y., Wang H., Zhuang X.Y., Xu M., Zhou J. (2021). Charge-Gradient Hydrogels Enable Direct Zero Liquid Discharge for Hypersaline Wastewater Management. Adv. Mater..

[4] Hadden W.J., Young J.L., Holle A.W., McFetridge M.L., Kim D.Y., Wijesinghe P., Taylor-Weiner H., Wen J.H., Lee A.R., Bieback K. (2017). Stem cell migration and mechanotransduction on linear stiffness gradient hydrogels. Proc. Natl. Acad. Sci. USA.

[5] Zhu D.Q., Trinh P., Li J.F., Grant G.A., Yang F. (2021). Gradient hydrogels for screening stiffness effects on patient-derived glioblastoma xenograft cellfates in 3D. J. Biomed. Mater. Res. Part A.

[6] Xu P.P., Tan Y., Wang X.L., Xu H.X., Wang D., Yang Y., An W.L., Xu S.M. (2020). Multidimensional gradient hydrogel and its application in sustained release. Colloid Polym. Sci..

[7] Liu X., Liu S., Yang R., Wang P.H., Zhang W.J., Tan X.Y., Ren Y.H., Chi B. (2021). Gradient chondroitin sulfate/poly (gamma-glutamic acid) hydrogels inducing differentiation of stem cells for cartilage tissue engineering. Carbohydr. Polym..

[8] Liu E., Zhu D.Q., Diaz E.G., Tong X.M., Yang F. (2021). Gradient Hydrogels for Optimizing Niche Cues to Enhance Cell-Based Cartilage Regeneration. Tissue Eng. Part A.

[9] Zinkovska N., Smilek J., Pekar M. (2020). Gradient Hydrogels-The State of the Art in Preparation Methods. Polymers.

[10] Gharazi S., Zarket B.C., DeMella K.C., Raghavan S.R. (2018). Nature-Inspired Hydrogels with Soft and Stiff Zones that Exhibit a 100-Fold Difference in Elastic Modulus. ACS Appl. Mater. Interfaces.

[11] Cho K., Lee H.J., Han S.W., Min J.H., Park H., Koh W.G. (2015). Multi-Compartmental Hydrogel Microparticles Fabricated by Combination of Sequential Electrospinning and Photopatterning. Angew. Chem. Int. Ed..

[12] Cross L.M., Shah K., Palani S., Peak C.W., Gaharwar A.K. (2018). Gradient nanocomposite hydrogels for interface tissue engineering. Nanomedicine.

[13] Li C.C., Ouyang L.L., Pence I.J., Moore A.C., Lin Y.Y., Winter C.W., Armstrong J.P.K., Stevens M.M. (2019). Buoyancy-Driven Gradients for Biomaterial Fabrication and Tissue Engineering. Adv. Mater..

[14] Fan W.X., Shan C.Y., Guo H.Y., Sang J.W., Wang R., Zheng R.R., Sui K.Y., Nie Z.H. (2019). Dual-gradient enabled ultrafast biomimetic snapping of hydrogel materials. Sci. Adv..

[15] Kim C., Young J.L., Holle A.W., Jeong K., Major L.G., Jeong J.H., Aman Z.M., Han D.W., Hwang Y., Spatz J.P. (2020). Stem Cell Mechanosensation on Gelatin Methacryloyl (GelMA) Stiffness Gradient Hydrogels. Ann. Biomed. Eng..

[16] Gorgieva S., Kokol V. (2015). Processing of gelatin-based cryogels with improved thermomechanical resistance, pore size gradient, and high potential for sustainable protein drug release. J. Biomed. Mater. Res. Part A.

[17] Canadas R.F., Patricio P., Brancato V., Gasperini L., Caballero D., Pires R.A., Costa J.B., Pereira H., Yong P., da Silva L.P. (2020). Convection patterns gradients of non-living and living micro-entities in hydrogels. Appl. Mater. Today.

[18] Ko H., Suthiwanich K., Mary H., Zanganeh S., Hu S.K., Ahadian S., Yang Y.Z., Choi G., Fetah K., Niu Y.T. (2019). A simple layer-stacking technique to generate biomolecular and mechanical gradients in photocrosslinkable hydrogels. Biofabrication.

[19] Shao Z.J., Wu S.S., Zhang Q., Xie H., Xiang T., Zhou S.B. (2021). Salt-responsive polyampholyte-based hydrogel actuators with gradient porous structures. Polym. Chem..

[20] Kaberova Z., Karpushkin E., Nevoralová M., Vetrík M., Šlouf M., Dušková-Smrčková M. (2020). Microscopic Structure of Swollen Hydrogels by Scanning Electron and Light Microscopies: Artifacts and Reality. Polymers.

[21] Scaffaro R., Lopresti F., Botta L., Rigogliuso S., Ghersi G. (2016). Preparation of three-layered porous PLA/PEG scaffold: Relationship between morphology, mechanical behavior and cell permeability. J. Mech. Behav. Biomed. Mater..

[22] Tan Y., Wang D., Xu H.X., Yang Y., Wang X.L., Tian F., Xu P.P., An W.L., Zhao X., Xu S.M. (2018). Rapid Recovery Hydrogel Actuators in Air with Bionic Large-Ranged Gradient Structure. ACS Appl. Mater. Interfaces.

[23] Xu G., Ding Z.Z., Lu Q., Zhang X.Y., Zhou X.Z., Xiao L.Y., Lu G.Z., Kaplan D.L. (2020). Electric field-driven building blocks for introducing multiple gradients to hydrogels. Protein Cell.

[24] Mredha M.T.I., Le H.H., Tran V.T., Trtik P., Cui J.X., Jeon I. (2019). Anisotropic tough multilayer hydrogels with programmable orientation. Mater. Horiz..

[25] Shi D.J., Shen J.L., Zhang Z.Y., Shi C., Chen M.Q., Gu Y.L., Liu Y. (2019). Preparation and properties of dopamine-modified alginate/chitosan-hydroxyapatite scaffolds with gradient structure for bone tissue engineering. J. Biomed. Mater. Res. Part A.

[26] Su C., Su Y.L., Li Z.Y., Haq M.A., Zhou Y., Wang D.J. (2017). In situ synthesis of bilayered gradient poly(vinyl alcohol)/hydroxyapatite composite hydrogel by directional freezing-thawing and electrophoresis method. Mater. Sci. Eng. C.

[27] Xu P.P., Xu H.X., Yang Y., Wang X.L., An W.L., Hu Y., Xu S.M. (2020). A nonswellable gradient hydrogel with tunable mechanical properties. J. Mater. Chem. B.

[28] Tan Y., Xu S.M., Wu R.L., Du J., Sang J.L., Wang J.D. (2017). A gradient Laponite-crosslinked nanocomposite hydrogel with anisotropic stress and thermo-response. Appl. Clay Sci..

[29] Guo K.C., Zhu W.Z., Wang J., Sun W., Zhou S., He M. (2021). Fabrication of gradient anisotropic cellulose hydrogels for applications in micro-strain sensing. Carbohydr. Polym..

