# Supplementary material for: Gradient Hydrogels—Overview of Techniques Demonstrating the Existence of a Gradient
Source: Polymers (Basel). 2022 Feb 23;14(5):866. doi: 10.3390/polym14050866 (PMC8912830; doi:10.3390/polym14050866)
Supplement: Supplementary file 1 [file polymers-14-00866-s001.zip › polymers-1591189 supplyment and non published material/polymers-1591189 non published material 1/Gharazi_permission.pdf]

RightsLink®

Home | Help | Email Support | Natalia Zinkovska

ACS Publications
Most Trusted. Most Cited. Most Read.

Nature-Inspired Hydrogels with Soft and Stiff Zones that Exhibit a 100-Fold Difference in Elastic Modulus

Author: Salimeh Gharazi, Brady C. Zarket, Kerry C. DeMella, et al
Publication: Applied Materials
Publisher: American Chemical Society
Date: Oct 1, 2018

Copyright © 2018, American Chemical Society

PERMISSION/LICENSE IS GRANTED FOR YOUR ORDER AT NO CHARGE

This type of permission/license, instead of the standard Terms and Conditions, is sent to you because no fee is being charged for your order. Please note the following:

- Permission is granted for your request in both print and electronic formats, and translations.
- If figures and/or tables were requested, they may be adapted or used in part.
- Please print this page for your records and send a copy of it to your publisher/graduate school.
- Appropriate credit for the requested material should be given as follows: "Reprinted (adapted) with permission from {COMPLETE REFERENCE CITATION}. Copyright {YEAR} American Chemical Society." Insert appropriate information in place of the capitalized words.
- One-time permission is granted only for the use specified in your RightsLink request. No additional uses are granted (such as derivative works or other editions). For any uses, please submit a new request.

If credit is given to another source for the material you requested from RightsLink, permission must be obtained from that source.

BACK CLOSE WINDOW

© 2021 Copyright - All Rights Reserved | Copyright Clearance Center, Inc. | Privacy statement | Terms and Conditions
Comments? We would like to hear from you. E-mail us at customercare@copyright.com
