# Supplementary material for: Gradient Hydrogels—Overview of Techniques Demonstrating the Existence of a Gradient
Source: Polymers (Basel). 2022 Feb 23;14(5):866. doi: 10.3390/polym14050866 (PMC8912830; doi:10.3390/polym14050866)
Supplement: Supplementary file 1 [file polymers-14-00866-s001.zip › polymers-1591189 supplyment and non published material/polymers-1591189 supplyment/Table_S2_Overview_characterization_techniques.pdf]

Characterization technique	Measured parameters	Gradient type	Merits/Limitations
XPS	elemental distribution of the hydrogel surface<br>- element ratios/mole fractions of monomer<br>- cross-linking density<br>qualitative + quantitative technique	Chemical + physical	− surface characterization<br>− preliminary pretreatment of the samples<br>(applies only for the lyophilized samples)
FLUO	characterization of inner environment or structural changes using fluorescent probes<br>qualitative + quantitative technique	Chemical	+ applies in "wet" state<br>+ possible tracking of gradient formation in real time<br>−sensitive to pH, ionic strength and temperature changes<br>+ applies in "wet" state<br>− preliminary pretreatment of the sample<br>(labelling by hydrophilic fluorescent probe)
RAMAN	chemical structure and identity,<br>phase and polymorphism in high-water content media<br>- cross-linking density<br>qualitative + quantitative technique	Chemical + physical	+ insensitive to aqueous absorption bands<br>+ doesn't need sample preparation<br>− large fluorescence background<br>(can suppress the smaller Raman signals)
CLSM	three-dimensional imaging of samples (by selectively accessing the fluorescence signals from different planes)<br>-distribution of functional groups labelled by fluorescent dye<br>- cross-linking density	Chemical + physical	− preliminary pretreatment of the sample<br>(labelling by fluorescent probe)<br>− limited depth of field<br>+ applies in "wet" state
FTIR	presence of the specific chemical/functional groups or inorganic compounds	Chemical + physical	− sensitive to aqueous absorption bands<br>− preliminary pretreatment of the samples

	(alterations in characteristic patterns of absorption bands indicate change in the material composition)<br>qualitative technique		(applies for the lyophilized samples or samples dissolved in organic solvent)

Characterization technique	Measured parameters	Gradient type	Merits/Limitations
SEM	imaging of hydrogel surface and internal structure<br>- pore sizes, shapes and distribution<br>- pore interconnectivity<br>-cross-linking density<br>- wall thickness<br>- fiber orientation<br>qualitative technique	Physical	+ high resolution (in contrast to<br>− preliminary pretreatment of the samples<br>(applies only for the lyophilized samples)<br>− structural artifacts<br>(freeze-drying process could cause changes in morphology)
POM	composition and three-dimensional structure<br>- direction of polymer alignment/orientation of rigid molecules based on optical anisotropy of the material<br>- absorption color and optical path boundaries between layers<br>qualitative technique	Physical	− anisotropic hydrogels
micro-CT	3D imaging of internal structure of hydrogels<br>- porosity<br>- cross-linking density<br>- wall thickness	Physical	+ 3D volume reconstruction<br>− demand labeling or contrasting the hydrogel chemically<br>− limited resolution<br>− preliminary pretreatment of the samples

	- fiber orientation<br>qualitative technique		(applies for the lyophilized samples
AFM	imaging of hydrogel topography<br>- roughness<br>- pore sizes, shapes and distribution<br>- wall thickness<br>qualitative + quantitative technique	Physical	− surface characterization<br>+ applies in “wet” state
