# Supplementary material for: Gradient Hydrogels—Overview of Techniques Demonstrating the Existence of a Gradient
Source: Polymers (Basel). 2022 Feb 23;14(5):866. doi: 10.3390/polym14050866 (PMC8912830; doi:10.3390/polym14050866)
Supplement: Supplementary file 1 [file polymers-14-00866-s001.zip › polymers-1591189 supplyment and non published material/polymers-1591189 supplyment/Table_S3_List of abbreviations.pdf]

**Abbreviation**	**Meaning**
XPS	X-ray photoelectron spectroscopy
FLUO	Fluorescence spectroscopy
HAp	hydroxyapatite microparticles
GG	gellun gum
FITC	fluorescein isothiocyanate
FTIR	Fourier transform infrared spectroscopy
SEM	Scanning electron microscopy
GelMA-MκCA	methacrylated kappa-carrageenan
PLA	polylactic acid
PEG	polyethylene glycol
HEA	hydroxyethyl acrylate
NIPAM	N-isopropylacrylamide
AFM	Atomic force microscopy
XRD	X-ray powder diffraction
POM	Polarizing optical microscopy
micro-CT	Computer microtomography
MPTMS	organic–inorganic monomer [3-(methacryloyloxy)-propyl]trimethoxy-silane
DMAA	*N,N′*-dimethylacrylamide
LAP	Laponite microparticles
NC	nanocomposite
