# Supplementary figures and images for: Gradient Hydrogels—Overview of Techniques Demonstrating the Existence of a Gradient
Source: Polymers (Basel). 2022 Feb 23;14(5):866. doi: 10.3390/polym14050866 (PMC8912830; doi:10.3390/polym14050866)

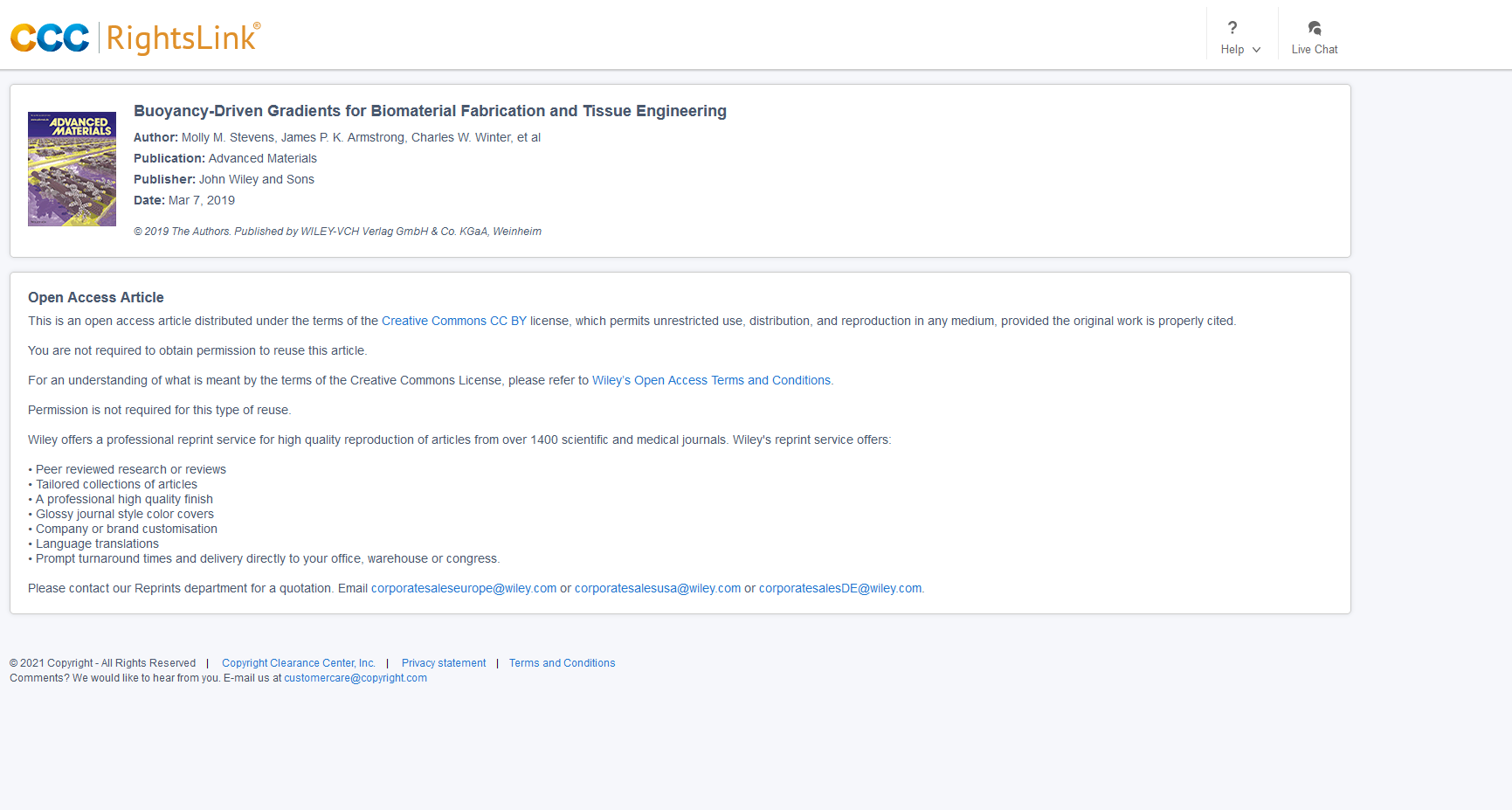

Supplement: Supplementary file 1 [file polymers-14-00866-s001.zip › polymers-1591189 supplyment and non published material/polymers-1591189 non published material 1/Li_1A_permission.png]

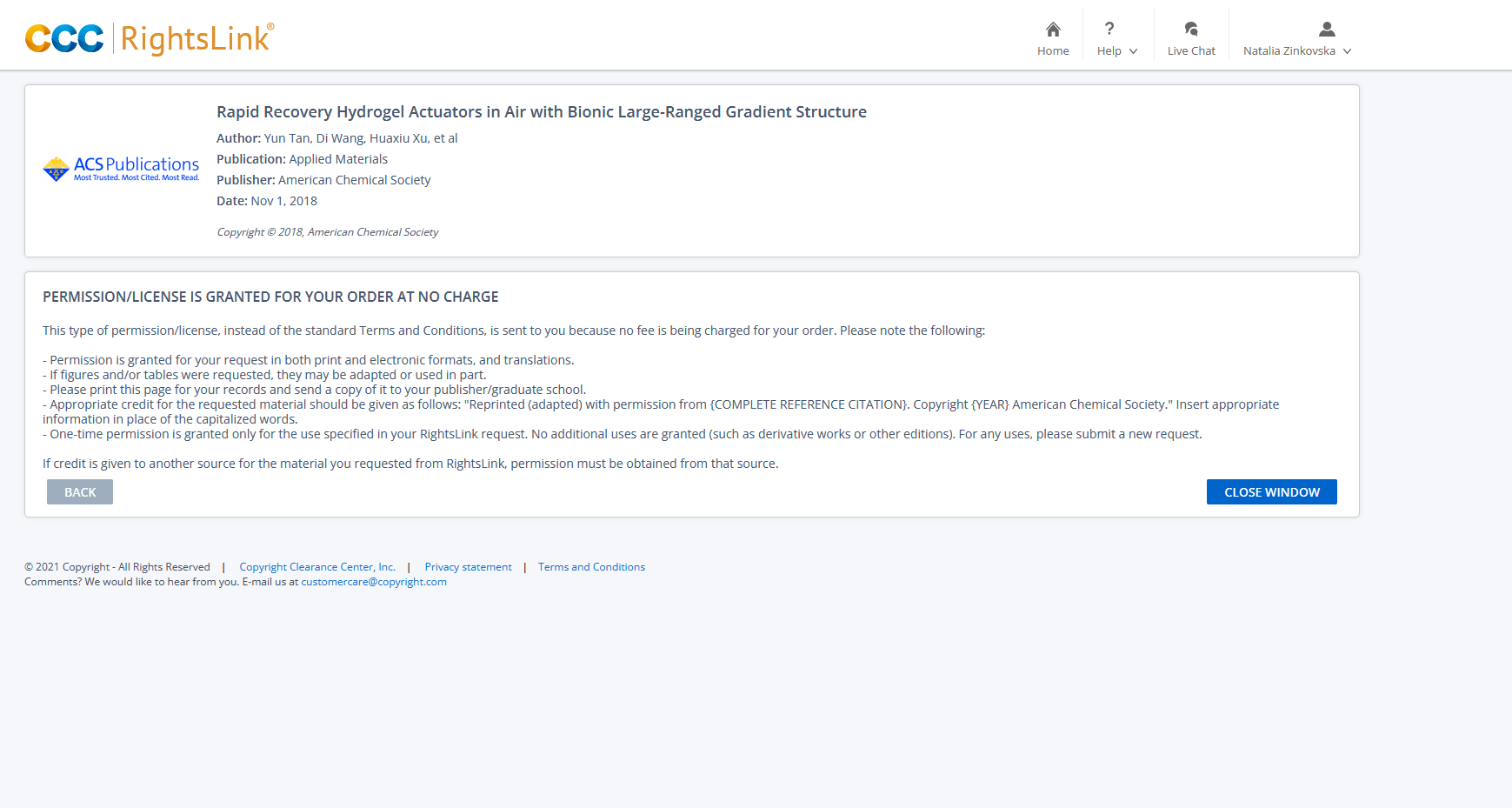

Supplement: Supplementary file 1 [file polymers-14-00866-s001.zip › polymers-1591189 supplyment and non published material/polymers-1591189 non published material 1/Tan_2018_permission.png]
